# Quantitative BOLD imaging at 3T: Temporal changes in hepatocellular carcinoma and fibrosis following oxygen challenge

**DOI:** 10.1002/jmri.25189

**Published:** 2016-02-19

**Authors:** Andrew J. Patterson, Andrew N. Priest, David J. Bowden, Tess E. Wallace, Ilse Patterson, Martin J. Graves, David J. Lomas

**Affiliations:** ^1^ Department of RadiologyUniversity of Cambridge and Cambridge University Hospitals NHS Foundation TrustHills RoadCambridgeCB2 0QQUK

**Keywords:** BOLD, liver, hepatocellular carcinoma, fibrosis

## Abstract

**Purpose:**

To evaluate the utility of oxygen challenge and report on temporal changes in blood oxygenation level‐dependent (BOLD) contrast in normal liver, hepatocellular carcinoma (HCC) and background fibrosis.

**Materials and Methods:**

Eleven volunteers (nine male and two female, mean age 33.5, range 27–41 years) and 10 patients (nine male and one female, mean age 68.9, range 56–87 years) with hepatocellular carcinoma on a background of diffuse liver disease were recruited. Imaging was performed on a 3T system using a multiphase, multiecho, fast gradient echo sequence. Oxygen was administered via a Hudson mask after 2 minutes of free‐breathing. Paired *t*‐tests were performed to determine if the mean pre‐ and post‐O_2_ differences were statistically significant.

**Results:**

In patients with liver fibrosis (*n* = 8) the change in $T_{2}^{*}$ following O_2_ administration was elevated (0.88 ± 0.582 msec, range 0.03–1.69 msec) and the difference was significant (*P* = 0.004). The magnitude of the BOLD response in patients with HCC (*n* = 10) was larger, however the response was more variable (1.07 ± 1.458 msec, range –0.93–3.26 msec), and the difference was borderline significant (*P* = 0.046). The BOLD response in the volunteer cohort was not significant (*P* = 0.121, 0.59 ± 1.162 msec, range –0.81–2.44 msec).

**Conclusion:**

This work demonstrates that the BOLD response following oxygen challenge within cirrhotic liver is consistent with a breakdown in vascular autoregulatory mechanisms. Similarly, the elevated BOLD response within HCC is consistent with the abnormal capillary vasculature within tumors and the arterialization of the blood supply. Our results suggest that oxygen challenge may prove a viable BOLD contrast mechanism in the liver. J. Magn. Reson. Imaging 2016;44:739–744.

Hepatocellular carcinoma (HCC) is the fifth most common cancer and the third leading cause of cancer‐related death worldwide.^1^ The major clinical risk factors are underlying chronic liver disease, typically secondary to viral infections, nonalcoholic steatohepatitis, or chronic alcoholic liver disease. Men are most commonly affected, with the mean age of presentation ∼60 years.^2^

Several studies have investigated the utility of blood oxygenation level‐dependent (BOLD) contrast changes in measuring oxygen perturbation within tumors. In 1997 Griffiths et al demonstrated that hyperoxic and hypercapnic gas mixtures can change the deoxyhemoglobin fraction of blood and act as a viable endogenous contrast mechanism in a variety of tumor etiologies.^3^ Subsequent studies have continued to hypothesize that autoregulatory function is compromised in tumors as a result of chaotic vasculature and abnormal smooth muscle cell development. The literature has focused on carbogen (95% O_2_ and 5% CO_2_)^3^, ^4^ studies, thought to increase vasodilation within healthy tissues, which alters the deoxy/oxyhemoglobin fraction and induces BOLD signal intensity change in healthy tissue. BOLD signal changes in response to carbogen are thought to be more consistent than oxygen; however, carbogen studies have reported up to 30% aborted scans owing to subjects experiencing “air hunger.”^3^, ^4^ To mitigate this issue, investigators have evaluated the utility of medical‐grade oxygen to induce a BOLD response; for example, Hallac et al used oxygen and noted differences in BOLD responses between cervical tumors and healthy tissue.^5^

HCC is often present on a background of diffuse liver disease; however, only one study to date has explicitly investigated BOLD response as a function of liver fibrosis. Jin et al used steady‐state and dynamic carbogen challenges with different diethylnitrosamine‐induced liver fibrosis stages to depict hemodynamic alterations during disease progression in a rat model.^6^ They found that both mean Δ $R_{2}^{*}$ (*r* = –0.773) and area of liver activation (*r* = –0.691) were inversely correlated with quantitative histopathological assessment of the percentage liver fibrosis (*P* < 0.001). This result warrants further investigation into BOLD contrast as a marker of hepatic fibrosis.

The purpose of this study was to investigate the utility of oxygen challenge at inducing a BOLD response in the liver. This study reports the observed temporal changes in $T_{2}^{*}$ in healthy volunteers and compares this to patients with HCC and background diffuse liver disease.

## Materials and Methods

### Study Design

This prospective single‐center study was conducted following local Research Ethics committee approval. All participants gave informed written consent. Eleven healthy volunteers (nine male and two female, mean age 33.5, range 27–41 years) with no history of hepatobiliary or cardiovascular disease and 10 patients (nine male and one female, mean age 68.7, range 56–87 years) with a recent diagnosis of HCC were recruited. Four patients were diagnosed with HCC after biopsy and the remaining six were diagnosed using imaging following the EASL criteria (European Association for Study of Liver).^7^ Eight out of 10 patients had background liver fibrosis, which was diagnosed after biopsy (*n* = 7) or using imaging criteria (*n* = 3). The underlying etiologies were established following multidisciplinary review of biopsy, blood, and imaging data. All participants fasted for a minimum of 8 hours prior to the examination and had not undergone any tumor treatment.

### Imaging Protocol

**Figure 1:**
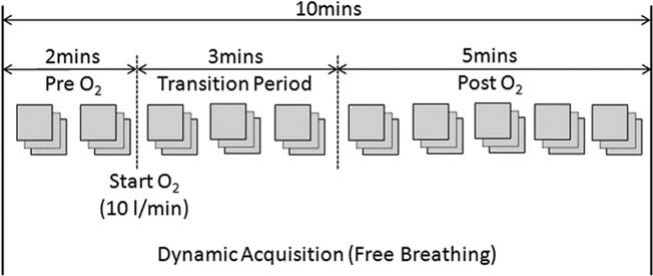
BOLD protocol: dynamic multiphase multiecho free‐breathing respiratory‐triggered acquisition acquired pre‐ and post‐O_2_. Mean pre‐O_2_ and mean post‐O_2_ $T_{2}^{*}$ values were compared after allowing a 3‐minute transition period for the response to equilibrate.

All the examinations were performed on a 3T whole‐body magnetic resonance imaging (MRI) system (Signa HDx, GE Healthcare, Waukesha, WI). Participants were imaged feet first, supine, using an 8‐channel cardiac receive coil. A multiecho fast gradient echo sequence was prescribed in a sagittal plane centered on the tumor. The sequence acquired five slices each with 10 separate echoes, with the following parameters: echo times (TEs) = 2.3/6.9/11.5–43.7 msec; repetition time (TR) = 46 msec; flip angle = 15°; matrix size = 192 × 96; ASSET factor = 2; field of view (FOV) = 35 cm; slice thickness = 8 mm. The BOLD sequence was modified to enable multiecho, multiphase operation with respiratory triggering using abdominal bellows. Each slice was acquired in 2.3 seconds. The BOLD protocol consisted of a 10‐minute free‐breathing respiratory‐triggered acquisition. Medical‐grade oxygen (BOC Healthcare, UK) was administered via a Hudson mask at 10 L/min after 2 minutes of free‐breathing room air (Fig. 1). The mask was left on for the duration of the scan. A dynamic contrast‐enhanced acquisition was also performed at the same slice location.

### Image Analysis

In‐house software was developed using MatLab (v. 8.3, MathWorks, Natick, MA) to compute voxel‐wise $T_{2}^{*}$ maps from the multiecho images at each temporal phase. The nonlinear Levenberg–Marquardt algorithm was used to compute $T_{2}^{*}$ values after initially seeding the fit with a log‐linear approximation.

To account for residual differences in diaphragm position in the respiratory‐triggered acquisition, an affine transform was applied to register each temporal phase. A rectangular mask was used so that the rigid registration algorithm was optimal over the area of interest. The image registration was performed by developing bespoke software in C++ that utilized the Insight Toolkit (www.itk.org).

Regions of interest (ROIs) were defined on the spatially registered maps using in‐house software developed in MatLab. ROIs were defined to encompass background liver while excluding major blood vessels and to delineate the hepatocellular carcinoma. ROIs were defined by a radiologist (D.J.B.) with 4 years experience after comparing comatched contrast enhanced images. The radiologist also recorded the maximum diameter of each tumor.

### Statistical Analysis

Mean $T_{2}^{*}$ pre‐O_2_ was computed by averaging the temporal phases prior to O_2_ administration. Similarly, the mean $T_{2}^{*}$ post‐O_2_ was computed by averaging the temporal phases during O_2_ breathing, acquired after a 3‐minute delay to allow the BOLD effect to equilibrate. Normality assumptions were formally assessed using a Shapiro–Wilk's test. Paired *t*‐tests were subsequently performed to establish whether the differences between pre‐ and post‐O_2_ $T_{2}^{*}$ values were significant. Student's *t*‐tests were also used to compare native (pre‐O_2_) $T_{2}^{*}$ in tumors against diffuse liver disease and healthy liver. *P* < 0.05 was defined as statistically significant. The statistical analysis was performed using the R programming language (v. 3.1.1, R Foundation for Statistical Computing, Vienna, Austria).

## Results

Etiologies for the patient cohort included nonalcoholic steatohepatitis (*n* = 4), alcohol‐related cirrhosis (*n* = 1), cirrhosis following alpha‐1 antitrypsin deficiency (*n* = 1), hepatitis B (*n* = 1), and hepatitis C (*n* = 1); one patient developed a spontaneous HCC and in one patient the etiology is unknown. The mean of the maximum tumor diameters was 4.4 cm (range 1.8–7.5 cm).

All the volunteers and patients completed the BOLD experiment. No datasets were excluded due to image quality issues.

**Figure 2:**
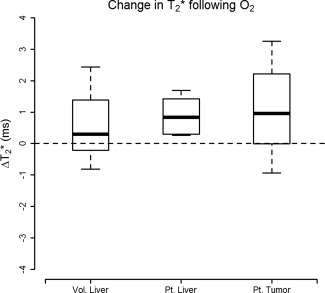
Change in $T_{2}^{*}$ following the administration of O_2_ illustrates comparative response in healthy liver tissue in normal volunteers against patients with diffuse liver disease and within tumor tissue. The boxplots illustrate how response in BOLD following O_2_ in diseased liver was consistently elevated (0.88 ± 0.58, *P* = 0.004); the mean response within HCC was larger, however, it was also more variable (1.07 ± 1.46, *P* = 0.046).

The change in $T_{2}^{*}$ following the administration of O_2_ is summarized in Fig. 2 and Table 1. The change in $T_{2}^{*}$ in patients with confirmed liver fibrosis (*n* = 8) was consistently elevated 0.88 ± 0.582 msec, range (0.03–1.69 msec), and the difference in $T_{2}^{*}$ following O_2_ was statistically significant (*P* = 0.004). The response in tumor tissue (*n* = 10) was larger but also more variable 1.07 ± 1.458 msec (range –0.93–3.26 msec); the difference was borderline statistically significant (*P* = 0.046). The change in $T_{2}^{*}$ was also positive following O_2_ administration in healthy volunteers (*n* = 11, 0.59 ± 1.162 msec, range –0.81–2.44 msec); however, the change was not significant (*P* = 0.121).

**Table 1:** Change in $T_{2}^{*}$ in Response to O_2_

		$T_{2}^{*}$ (msec)			
		Pre O_2_	Post O_2_	Post‐Pre O_2_	*P*‐value
Patients	Liver fibrosis (*n* = 8)	15.8 ± 6.40	16.7 ± 6.77	0.88 ± 0.582	0.004
	HCC (*n* = 10)	23.7 ± 7.52	24.8 ± 6.87	1.07 ± 1.458	0.046
Volunteers	Healthy liver (*n* = 11)	17.1 ± 3.71	17.7 ± 4.80	0.59 ± 1.162	0.121

Mean ± SD.

**Figure 3:**
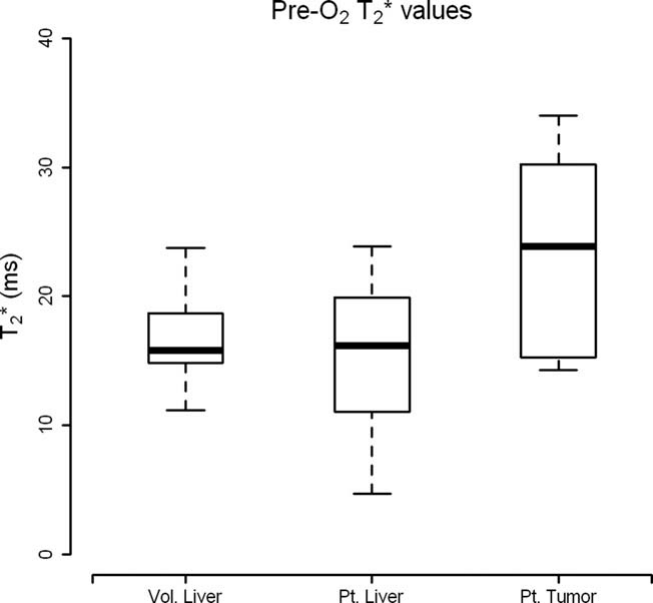
Absolute native $T_{2}^{*}$ values (pre‐O_2_) that illustrate how $T_{2}^{*}$ within HCC is significantly elevated with respect to healthy liver (*P* = 0.025) and with respect to their diseased background liver (*P* = 0.01).

**Figure 4:**
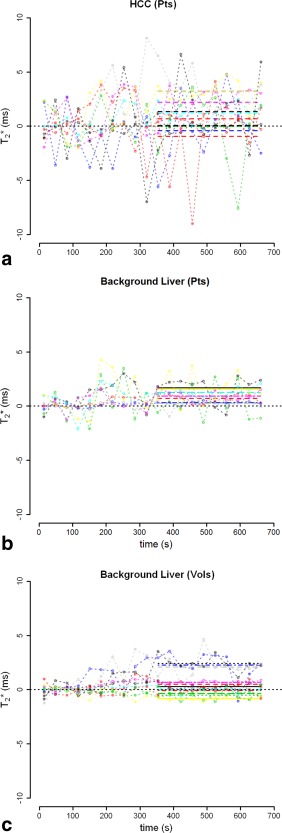
Temporal BOLD changes in $T_{2}^{*}$ following O_2_ administration. Change is $T_{2}^{*}$ is plotted relative to the mean baseline signal. The best‐fit line is shown for the response post‐O_2_ following a 3‐minute transition period.

$T_{2}^{*}$ of tumor pre‐O_2_ (23.7 ± 7.52 msec) was elevated with respect to the patients' background liver (15.2 ± 5.84 msec), *P* = 0.01, and with respect to the volunteer liver (17.1 ± 3.71 msec), *P* = 0.025 (Fig. 3). The response to O_2_ is shown on a per‐subject basis in Fig. 4.

## Discussion

This study demonstrated the feasibility of assessing BOLD contrast changes at 3T in response to oxygen challenge in healthy and chronic diffuse liver disease, and within HCC. We report a statistically significant elevation in $T_{2}^{*}$ following O_2_ administration in humans with confirmed liver fibrosis. We also report elevated changes in HCC consistent with Bane et al's recent study that reported the feasibility and test–retest variability of BOLD response in HCC using both O_2_ and carbogen challenge at 1.5 and 3T.^8^

In volunteers, no statistically significant change was seen in response to 100% oxygen breathing, which is consistent with previous reports measuring oxygen‐induced $T_{2}^{*}$ changes in the liver.^9^, ^10^, ^11^ Qualitatively, we observed that eight responses remained close to the baseline signal, with $T_{2}^{*}$ values in three volunteers appearing to elevate slowly over time. Varying $T_{2}^{*}$ responses to 100% oxygen challenge have been reported in other healthy tissue types, indicating that hyperoxia‐induced changes in perfusion and oxygenation are organ‐specific.^11^ Blood flow to the liver is unique, given its high vascularity and dual blood supply from the portal vein and hepatic artery. Approximately 75% of total hepatic blood flow is partially deoxygenated venous blood supplied by the portal vein,^12^ which conducts blood from the stomach, pancreas, small intestine, and spleen to the liver. Increases in dissolved oxygen in arterial blood may therefore not be completely transmitted through portal circulation to the liver; rather, the O_2_ status of the liver is partly dictated by oxygen extraction in the gastrointestinal tract.^13^ Together, these factors may explain the negligible change in $T_{2}^{*}$ with hyperoxia observed in healthy volunteers. Other studies have shown that breathing pure oxygen may induce a slight vasoconstriction in vessels,^14^ which coupled with a small increase in the oxygenated hemoglobin fraction of venous blood may cause the deoxygenated hemoglobin fraction within each imaging voxel to decrease. This could account for the modest $T_{2}^{*}$ increase seen in three of our subjects.

Physiological response to carbogen differs from response to 100% oxygen, as CO_2_ is a potent vasodilator. In healthy liver tissue, perfusion is significantly increased in response to hypercapnia.^13^, ^15^ Tissue oxygenation generally improves from augmented blood flow, but as most of the increase occurs in the portal vein, there is an offset in hepatic arterial flow, leading to an overall increase in the ratio of deoxyhemoglobin to oxyhemoglobin. Highly significant increases in $R_{2}^{*}$ (=1/ $T_{2}^{*}$) have been found in healthy liver in response to carbogen breathing,^10^, ^11^, ^15^ indicating carbogen may be a useful stimulus. Bane et al recently performed a systematic comparison of BOLD response in HCC comparing oxygen and carbogen and found an elevated BOLD response within HCC following oxygen challenge; however, the response to carbogen was not significantly elevated.^8^ This suggests that the optimal stimuli for BOLD liver examinations may need to be tailored depending on the pathology of interest.

With the evolution of hepatic fibrosis, progressive disruption of normal liver architecture is seen, which gives rise to regional and global changes in perfusion.^16^ Venous flow typically decreases due to increased resistance in the portal venous system, and may bypass the parenchyma completely via portosystemic venous shunts.^16^ Hepatic arterial flow commonly increases to counteract this effect. Impaired hemodynamic response and increased arterial blood flow to the liver may account for the elevated $T_{2}^{*}$ response seen in diseased liver tissue. This is consistent with a breakdown in autoregulatory function, and is similar to the reduced hemodynamic response to carbogen challenge observed by Jin et al in their rat model of fibrosis.^6^

Measuring native $T_{2}^{*}$ or change in $T_{2}^{*}$ (Δ $T_{2}^{*}$) in response to a hyperoxic stimulus has been proposed as an intrinsic marker of tumor oxygenation,^17^ as $T_{2}^{*}$ is proportional to the concentration of deoxyhemoglobin, which in turn relates to arterial blood pO_2_. In this study we observed a larger but more variable response to oxygen challenge in HCC, which may be expected given variations in oxygenation and perfusion, even within the same tumor type. The initial pathogenesis of HCC involves an increased arterial supply; however, in late‐stage HCC the arterial blood supply may decrease.^18^ This variability in blood supply, and the consequent proportions of oxyhemoglobin, could be a source of variability in the observed BOLD response.

We also observed that native tumor $T_{2}^{*}$ (pre‐O_2_) was elevated with respect to both the patients' background liver and with respect to the volunteer liver. This supports observations that elevated baseline $T_{2}^{*}$ can discriminate between highly vascularized and avascular tumor types.^19^ However, as evidenced by Jhaveri et al's negative report of any correlation between BOLD contrast and increased microvascular invasion, this suggests that interpretation of the BOLD signal within tumors is complex.^20^

There were pragmatic issues noted when measuring $T_{2}^{*}$ within HCC. In some cases, proximity of the tumor to the lung could cause dephasing in the acquired images near the air–tissue boundary, the very effect we were trying to measure. In this study two of the HCC patients had tumors juxtapositional to lung. In this study we chose to include these cases to evaluate the mean effect of HCC in patients, given our sample size constraints.

Liver biopsy is the reference standard for diagnosis and staging chronic liver disease, but it is an invasive procedure with potential complications, and reproducibility is poor due to the heterogeneity of fibrosis, small sample size, and inter‐ and intraobserver variability in histologic assessment.^21^ Alternative MR‐based imaging techniques have been proposed to noninvasively detect hepatic fibrosis, including MR elastography,^22^ as well as conventional diffusion‐weighted MRI and contrast‐enhanced methods. These methods have been shown to have differing potential in their diagnostic utility in diagnosis and grading of fibrosis, based on increased parenchymal stiffness, restricted water diffusion, and associated alterations in perfusion patterns with disease progression. Each of these respective methods is at a different stage in terms of clinical acceptance; MR elastography shows particular promise, in that stiffness has been shown to linearly relate to fibrosis stage.^22^ In this context the relationship between BOLD and fibrosis is unclear, and subsequent studies will need to compare the BOLD response to histopathological assessment of fibrosis grade in humans.

BOLD, however, has the advantage that it is noninvasive and is based on an endogenous contrast mechanism. Breathing oxygen was well tolerated by all subjects. In this study we performed quantitative $T_{2}^{*}$ measurements, rather than simply measuring relative changes in $T_{2}^{*}$‐weighted signal intensity, which can be sensitive to blood flow via signal intensity enhancement from unsaturated spins flowing into the imaging plane. We also demonstrated that measuring hepatic $T_{2}^{*}$ is feasible at 3T using a respiratory‐triggered, free‐breathing protocol, which enables dynamic measurements over time. Measuring the BOLD effect at 3T should be more sensitive than 1.5T to small perturbations in oxygenation, given the greater change in $T_{2}^{*}$ for a proportional change in the deoxyhemoglobin fraction.

Limitations of this study include sample size constraints. Greater significance could have been achieved with a larger cohort; nevertheless, this exploratory study demonstrates the feasibility of oxygen challenge for evaluating healthy and diseased liver tissue and HCC. Lack of an age match control group may lead to some bias in the results, as volunteers were systematically younger than the patient cohort. Future studies should consider acquiring more baseline $T_{2}^{*}$ maps pre‐O_2_; this would allow quantification of contrast repeatability and normal physiological variation. Future studies should also consider applying novel approaches that utilize the BOLD phenomena to assess vasomotor response.^23^

In conclusion, the BOLD response to oxygen challenge is a promising method for noninvasive diagnosis and staging of chronic liver disease. The increase in $T_{2}^{*}$ observed within diffuse liver disease in response to hyperoxia is consistent with a breakdown in vascular autoregulatory mechanisms and an increased arterial fraction of flow. Similarly, the elevated BOLD response within HCC is consistent with the abnormal arterialized vasculature in tumors. Further studies are needed to evaluate the benefit of measuring BOLD response in HCC, particularly with respect to its potential role as a biomarker of outcome and treatment response.
